# Scaling Up Diarrhea Prevention and Treatment Interventions: A Lives Saved Tool Analysis

**DOI:** 10.1371/journal.pmed.1000428

**Published:** 2011-03-22

**Authors:** Christa L. Fischer Walker, Ingrid K. Friberg, Nancy Binkin, Mark Young, Neff Walker, Olivier Fontaine, Eva Weissman, Akanksha Gupta, Robert E. Black

**Affiliations:** 1 Johns Hopkins University Bloomberg School of Public Health, Department of International Health, Baltimore, Maryland, United States of America; 2 Health Section, Programme Division, United Nations Children's Fund (UNICEF), New York, New York, United States of America; 3 World Health Organization (WHO), Department of Child and Adolescent Health, Geneva, Switzerland; 4 Futures Institute, Glastonbury, Connecticut, United States of America; Institute of Child Health, United Kingdom

## Abstract

**Background:**

Diarrhea remains a leading cause of mortality among young children in low- and middle-income countries. Although the evidence for individual diarrhea prevention and treatment interventions is solid, the effect a comprehensive scale-up effort would have on diarrhea mortality has not been estimated.

**Methods and Findings:**

We use the Lives Saved Tool (*LiST*) to estimate the potential lives saved if two scale-up scenarios for key diarrhea interventions (oral rehydration salts [ORS], zinc, antibiotics for dysentery, rotavirus vaccine, vitamin A supplementation, basic water, sanitation, hygiene, and breastfeeding) were implemented in the 68 high child mortality countries. We also conduct a simple costing exercise to estimate cost per capita and total costs for each scale-up scenario. Under the ambitious (feasible improvement in coverage of all interventions) and universal (assumes near 100% coverage of all interventions) scale-up scenarios, we demonstrate that diarrhea mortality can be reduced by 78% and 92%, respectively. With universal coverage nearly 5 million diarrheal deaths could be averted during the 5-year scale-up period for an additional cost of US$12.5 billion invested across 68 priority countries for individual-level prevention and treatment interventions, and an additional US$84.8 billion would be required for the addition of all water and sanitation interventions.

**Conclusion:**

Using currently available interventions, we demonstrate that with improved coverage, diarrheal deaths can be drastically reduced. If delivery strategy bottlenecks can be overcome and the international community can collectively deliver on the key strategies outlined in these scenarios, we will be one step closer to achieving success for the United Nations' Millennium Development Goal 4 (MDG4) by 2015.

*Please see later in the article for the Editors' Summary*

## Abstract 2

Using the Lives Saved Tool (LiST) Christa Fischer-Walker and colleagues estimate that scale-up of diarrhea prevention and treatment interventions over 5 years in 68 high child mortality countries could avert nearly 5 million deaths.

## Editors' Summary

**Background:**

Diarrhea—passing three or more loose or liquid stools per day—kills about 1.5 million young children every year, mainly in low- and middle-income countries. It is the second leading cause of death in under-5-year olds and causes nearly one in five child deaths. Diarrhea, which can lead to life-threatening dehydration, is a common symptom of gastrointestinal infections. The viruses, bacteria and parasites that cause diarrhea spread through contaminated food or drinking water, and from person-to-person through poor hygiene and inadequate sanitation (unsafe disposal of human excreta). Interventions that prevent diarrhea include improvements in water supplies, sanitation and hygiene, the promotion of breastfeeding, vitamin A supplementation, and vaccination against rotavirus (a major cause of diarrhea). Treatments for diarrhea include oral rehydration salts (ORS), which prevent and treat dehydration, and zinc supplementation, which decreases the severity and duration of diarrhea, and antibiotics for dysentery.

**Why Was This Study Done?:**

Deaths from diarrhea in young children have declined markedly over the past 30 years. However, if diarrhea deaths are not reduced further, it is unlikely that Millennium Development Goal 4 (MDG4; one of the goals agreed by world leaders in 2000 to reduce poverty)—the reduction of child mortality by two-thirds of the 1990 level by 2015—will be reached. In 2009, UNICEF and the World Health Organization (WHO) proposed a new diarrhea reduction plan. Although the effect of individual interventions in this plan is established, the likely effect of the whole package on diarrhea mortality has not been estimated. Such information would be useful for health policy planning. In this study, the researchers use the Lives Saved Tool (*LiST*) to estimate the potential lives saved by scale-up of diarrhea prevention and treatment interventions in 68 high child mortality countries that together account for 95% of child deaths. *LiST* is a child survival modeling tool that uses country-level under-5 death rates and cause of death profiles to model the effects of changes in health intervention package coverage on deaths among children.

**What Did the Researchers Do and Find?:**

The researchers calculated 2010 (baseline) coverage values for seven prevention interventions (breastfeeding, vitamin A supplementation, hand washing with soap, improved sanitation, improved water source, better household water treatment, and rotavirus vaccination) and for three treatment interventions (ORS, zinc supplementation, and antibiotics for dysentery) from published data. They then used *LiST* to estimate the effect on diarrhea deaths of scaling up intervention coverage according to two scenarios. The “ambitious” scenario assumed a feasible increase in the coverage of all interventions from the baseline year to 2015 in 68 countries with high child mortality. The “universal” scenario assumed an increase to near 100% coverage for all the interventions. Diarrhea mortality was reduced by 78% and 92% by 2015 under the ambitious and universal scenarios, respectively. Over the 5 years of the scale-up, the universal scenario averted nearly 5 million deaths. The researchers also estimated that the additional costs in 2015 of personal prevention and treatment interventions would be US$0.80 per capita with universal coverage; the additional costs for these interventions and all sanitation and water interventions would be US$3.24 per capita.

**What Do These Findings Mean?:**

These findings suggest that, with currently available interventions, it should be possible to reduce diarrhea deaths substantially at a reasonable cost. As with all computer models, the accuracy of these findings depends on the data and assumptions fed into the model, which does not, for example, account for the difficulties that may be encountered in scaling up intervention coverage in hard to reach populations. Similarly, the estimated costs associated with the two scenarios do not include the resources required to strengthen health systems in developing countries so that they are able to sustain high coverage levels of diarrhea prevention and treatment interventions. Nevertheless, these findings suggest that child mortality due to diarrhea could be significantly reduced by 2015 provided the international community acts collectively to deliver these interventions. Most importantly, the potential 1.4 million lives saved in that year would bring MDG4 a step closer simply by implementing existing low cost and effective interventions.

**Additional Information:**

Please access these Web sites via the online version of this summary at http://dx.doi.org/10.1371/journal.pmed.1000428.

- The World Health Organization provides information on diarrhea (in several languages); its 2009 report with UNICEF Diarrhea: why children are still dying and what can be done, which includes the WHO/UNICEF treatment and prevention plan, can be downloaded from the Internet
- The children's charity UNICEF, which protects the rights of children and young people around the world, provides information on water, sanitation, and hygiene, and on diarrhea (in several languages)
- The United Nations Millennium Development Goals provides information on ongoing world efforts to reduce child mortality
- More details on LiST are available

## Introduction

Diarrhea remains a leading cause of morbidity and mortality among children under 5 y of age in low- and middle-income countries [1]. Diarrhea mortality has declined from an estimated 4.5 million deaths in the early 1980s to 1.3 million in 2008 with the advent of oral rehydration salts (ORS), the implementation of routine vitamin A supplementation and measles vaccine, improved sanitation, access to clean water, and hand washing, which are major risk factors for diarrhea incidence in many parts of the world [1],[2]. Given the availability of cost-effective prevention and treatment interventions, however, the number of deaths owing to diarrhea remains unacceptably high. Further reduction of diarrhea mortality is critical if the fourth United Nations' Millennium Development Goal (MDG4) —reduction of child mortality by two-thirds of the 1990 level (12.4 million deaths per year) — is to be achieved by 2015.

In 2009, UNICEF and WHO published a report on diarrhea that included a package of key diarrhea prevention and treatment interventions to reduce diarrhea morbidity and mortality. The complete package includes improving access to safe water, community-wide promotion of sanitation, routine rotavirus and measles immunization, vitamin A supplementation and promotion of breastfeeding, and treatment with ORS and zinc [3]. Although the full package of prevention and treatment interventions is based on solid evidence supporting individual interventions, the effect that a universal scale-up effort would have on diarrhea mortality has not been estimated.

The Lives Saved Tool (*LiST*) is designed to enable international agencies and country planners to estimate the effect of increasing coverage of selected intervention combinations, such as the UNICEF/WHO recommended interventions for diarrhea, on mortality. *LiST* utilizes country-specific cause of death profiles and the effect of selected interventions on cause-specific mortality, and thus generates country-specific estimates of mortality reductions [4].

Here, we present two scenarios for the scale-up of diarrhea prevention and treatment interventions from 2010 to 2015. We use *LiST* to estimate the potential lives saved if each scale-up scenario were implemented in the 68 “Countdown to 2015” countries. These 68 countries were prioritized by UNICEF and partners on the basis of high child mortality rates; together they represent more than 95% of child deaths [5]. These data are critical for program planners, funders, and policy and decision makers to better understand the potential impact on mortality when investing in diarrhea prevention and treatment at the country level.

## Methods

*LiST* is a child survival modeling tool that uses country level under-5 mortality rates and cause of death profiles, and models the effects of changes in coverage of interventions on overall and cause-specific mortality rates for children under 5 y of age (http://www.jhsph.edu/dept/ih/IIP/list/spectrum.html) [4],[6]. It is built into the Spectrum policy modeling system, which includes a demographic platform based on UN population data, HIV, and family planning inputs. As a public access tool, analyses such as these can be performed, repeated, or altered by researchers or program and policy leaders alike. The effectiveness of each of the diarrhea interventions incorporated into the *LiST* tool has been recently reviewed by the Child Health Epidemiology Reference Group (CHERG) [7]–[11] and as part of a universal and published review [12],[13]. The CHERG reviews go beyond previously published systematic reviews and meta-analyses, utilizing all available data to provide the best estimate for the effect of each intervention on diarrhea-specific mortality [6]. In previous exercises, the *LiST* tool has estimated mortality reductions due to coverage changes that have matched well to the measured changes in mortality [14],[15].

### Establishing Baseline Values for Cause of Death and Coverage of Interventions

For this exercise, we generated *LiST* models for each of the 68 priority countries to project potential reductions in diarrhea mortality [5]. We used standard country-level child mortality rates as published by the Interagency Group for Child Mortality Estimation and the cause of death profiles published by the CHERG [16],[17]. We used baseline intervention coverage values for improved water supply, household connection, and improved sanitation from a special analysis of WHO/UNICEF Joint Monitoring Programme for Water Supply and Sanitation (JMP) data [18]. For these interventions we assumed no change in coverage between most recent available data points (typically 2008) and 2010. Vaccination and vitamin A coverage levels were based on WHO/UNICEF immunization program data [19]. All other baseline intervention coverage data were based on the most recent Demographic Health Survey (DHS), Multiple Indicator Cluster Survey (MICS), or Malaria Indicator Survey (MIS). Coverage data were not available for selected indicators in Papua New Guinea and Democratic People's Republic of Korea; for the former, we used estimated average regional data for Southeast Asia for ORS and antibiotic use, and for the latter, estimated data from China were used for breastfeeding and antibiotic use (see Table S1 for values).

Ten interventions proven to reduce diarrheal mortality were modeled. The preventive interventions were breastfeeding, vitamin A supplementation, hand washing with soap, improved sanitation (which encompasses toilet facilities and disposal of waste), improved drinking-water source, treatment of water in the home, and rotavirus vaccination. The treatment interventions included were ORS and zinc as well as antibiotics for dysentery. To model “treatment of water in the home,” we assumed a value of 0.21 for the effect of this indicator on diarrhea incidence and mortality [20]. Current coverage of this indicator is not available for all countries and it would be inappropriate to assume that 100% of households currently need clean water. We used the percent of children living in households with piped water as a proxy for current coverage of a clean water supply, available for all countries from the JMP [18]. This is likely to generate a conservative estimate of the potential impact of treatment of water, as it is probable that some households with piped water also need treatment. We included antibiotics for dysentery, though this was not included in the recent UNICEF/WHO recommended package, because it remains an important tool for treating dysenteric diarrhea. Lastly, measles vaccination, which is included in the UNICEF/WHO recommended package, was not included because measles-related diarrheal deaths are attributed to measles directly. Baseline values are presented in Table 1 for all interventions except breastfeeding. Additional details with regard to assumptions about breastfeeding coverage can be found in Table S1.

**Table 1:** Baseline coverage values for all interventions except breastfeeding.

Country	Percentage of Children <5 y with Access to/Practicing				Percentage of Children with Diarrhea in Last 2 wk Who Were Treated			Percentage of Children Who Received	
	Improved Water^a^	Treated Water^b^	Improved Sanitation^a^	Hand-Washing^c^	ORS^d^	Antibiotics for Dysentery^e^	Zinc for Diarrhea Treatment^f^	Rotavirus Vaccination^g^	Vitamin A Supplementation^h^
Afghanistan	48	4	37	17	30	16	0	0	96
Angola	50	20	57	17	40	20	0	0	82
Azerbaijan	80	50	45	17	10	5	0	0	90
Bangladesh	80	6	51	17	77	22	23	0	97
Benin	75	12	12	17	23	12	0	0	52
Bolivia	86	77	25	17	29	31	0	0	45
Botswana	95	62	60	17	49	24	0	0	15
Brazil	97	91	80	17	56	25	0	71	0
Burkina Faso	76	4	11	17	17	31	0	0	100
Burundi	72	6	46	17	38	26	0	0	80
Cambodia	61	16	29	17	21	12	0	0	88
Cameroon	74	15	47	17	13	38	0	0	92
CAR	67	2	34	17	17	39	0	0	68
Chad	50	5	9	17	15	12	0	0	0
China	89	83	55	13	29	33	0	0	0
Congo	71	28	30	17	18	22	0	0	10
Cote d'Ivoire	80	40	23	17	14	19	0	0	90
Djibouti	92	72	56	17	49	43	0	0	86
Democratic People's Republic of Korea	100	77	59	17	35	33	0	0	85
Democratic Republic of the Congo	46	9	23	17	31	21	0	0	98
Egypt	99	92	94	17	34	73	1	0	68
Equatorial Guinea	43	6	51	17	36	18	0	0	0
Eritrea	61	9	14	17	45	22	0	0	49
Ethiopia	38	7	12	17	20	5	0	0	88
Gabon	87	43	33	17	25	24	0	0	0
Gambia	92	33	67	17	41	61	0	0	28
Ghana	82	17	13	3	45	33	0	0	24
Guatemala	94	81	81	17	30	15	0	0	20
Guinea	71	10	19	17	33	17	0	0	94
Guinea-Bissau	61	9	21	17	26	42	0	0	66
Haiti	63	12	17	17	40	5	0	0	34
India	88	22	31	42	26	13	0	0	53
Indonesia	80	23	52	17	35	33	0	0	86
Iraq	79	76	73	17	31	82	0	0	0
Kenya	59	19	31	17	29	22	0	0	27
Laos	57	20	53	17	31	52	0	0	69
Lesotho	85	19	12	17	42	27	0	0	38
Liberia	68	2	17	17	53	49	0	0	85
Madagascar	41	7	11	4	12	20	0	0	97
Malawi	80	7	56	17	63	30	0	0	95
Mali	56	12	36	17	14	7	0	0	97
Mauritania	49	22	26	17	22	24	0	0	87
Mexico	94	87	85	17	4	15	0	0	63
Morocco	81	58	69	17	23	17	0	0	43
Mozambique	47	8	17	17	49	15	0	0	83
Myanmar	71	6	81	17	45	18	0	0	94
Nepal	92	19	32	17	29	25	0	0	93
Niger	48	7	9	17	18	9	0	0	92
Nigeria	58	6	32	17	18	46	0	0	74
Pakistan	90	33	45	17	41	50	0	0	97
Peru	82	70	68	14	28	28	0	0	0
Philippines	91	48	76	17	42	36	0	0	86
Papua New Guinea	40	10	45	17	30	30	0	0	7
Rwanda	65	4	54	17	21	13	0	0	76
Senegal	69	38	51	23	15	20	0	0	90
Sierra Leone	49	6	13	17	52	45	0	0	12
Somalia	30	19	23	17	9	32	0	0	100
South Africa	91	67	77	17	40	32	0	0	39
Sudan	57	28	34	17	58	45	0	0	67
Swaziland	69	32	55	17	86	24	0	0	44
Tajikistan	70	40	94	17	48	41	0	0	na
Tanzania	54	8	24	13	54	22	0	0	93
Togo	60	6	12	17	11	26	0	0	64
Turkmenistan	71	45	95	17	47	50	0	0	0
Uganda	64	3	47	14	40	47	0	0	67
Yemen	62	28	52	17	33	38	0	0	47
Zambia	60	14	49	17	60	14	0	0	96
Zimbabwe	82	36	44	17	6	8	0	0	20

a Data from JMP 2010.

b Used piped water values from the JMP 2010 report.

c Estimates based on work Curtis et al. [31].

d Most recent Demographic Health Survey (DHS)/Multiple Indicator Cluster Survey (MICS). Defined as percent of children with diarrhea in the past 2 wk who were treated with ORS or prepackaged ORS solutions.

e Most recent DHS/MICS. Assumed to be the same as percent of children with symptoms suggestive of pneumonia treated with an antibiotic.

f Most recent DHS/MICS. Defined as percent of children with diarrhea in the past 2 wk treated with zinc. If these data were not collected in the survey, 0 was the default value.

g Estimates from WHO/UNICEF estimates of national immunization coverage [32].

h UNICEF 2008 value or most recent. Countries listed as na are considered not to be Vitamin A deficient according to the Lancet Nutrition series and were excluded from the analysis.

### Modeling Increased Coverage of Interventions

Within the model, all chosen interventions have a direct impact on diarrheal mortality reduction. Four of the seven interventions—improved water source, treatment of water in the home, hand washing with soap, and improved sanitation—also have an indirect impact on multiple causes of mortality via a reduction in the rate of stunting. *LiST* applies the documented effectiveness for each intervention to the total diarrheal deaths possible among children under 5 for each given year. For each intervention the effectiveness value is applied to the residual number of diarrheal deaths available to “save” for that year thus eliminating the potential to double count lives saved.

The scale-up scenarios presented here assume a linear increase in coverage from the baseline coverage year, 2010 (using the most recent data available) through the year 2015; this allowed us to generate the total number of diarrheal deaths, by country for each year between 2010 and 2015. For an estimate of baseline diarrheal deaths we applied the 2008 overall mortality rate and the cause of death structure to the 2010 population.

We applied two different scale-up scenarios of the seven diarrhea prevention and three treatment interventions, representing ambitious but feasible coverage objectives and a universal coverage plan. The first or “ambitious” scenario represents what is felt to be an essential and realizable scale-up strategy as countries strive to reduce under-five mortality in the context of achievement of MDG4, whereas the second or “universal” scenario represents maximum levels that could be achieved through an aggressive, highly concerted, and better-funded initiative. Table 2 shows the coverage levels used in the analysis, representing the ambitious and universal scale-up scenarios. For breastfeeding, coverage estimates vary by age group (<1 mo, 1–5 mo, 6–11 mo, 12–23 mo) and degree of exclusivity (exclusive, predominant, partial, none). For any country that has already attained the modeled level of coverage for a specific intervention, we assumed maintenance of the achieved coverage rate. Additional details on individual country calculations can be found in Table S1.

**Table 2:** Modeled target coverage rates by intervention for two scale-up plans for the 68 priority countries.

Intervention^a^	*LiST* Estimates of Effectiveness of Diarrheal Deaths Averted	Percent National Target Coverage among Children <5 y of Age by 2015	
		Ambitious Coverage	Universal Coverage
**ORS for treatment of all episodes**	93%^b^	75	90
**Zinc (10–14 d of supplementation) for treatment of all episodes**	23%	50	90
**Antibiotics for dysentery episodes**	99%^c^	75	90
**Rotavirus vaccine**	74%^d^	50	90
**Routine vitamin A supplementation for children 6–59 mo (twice yearly)**	32%	90	90
**Hand-washing with soap**	48%	35	55
**Improved sanitation (primarily toilet construction)**	69%	67	75
**Access to safe water (improving water quality at source and safe storage in home) ** [**18**]	17%	—	99
Africa	—	75	—
Asia	—	86	—
**Home purification of water**	21%	30	70
**Breastfeeding: RR of diarrhea mortality**			
Exclusive breastfeeding (no additional fluids or foods)			
0–5 mo	1.0	70	90
6–23 mo	1.0	—	—
Predominant breastfeeding (breastfeeding with only additional water and water based fluids)			
0–5 mo	2.28	10	5
6–23 mo	1.0	0	0
Partial breastfeeding (breastfeeding with additional fluids and/or foods)			
0–5 mo	4.62	10	0
6–11 mo	—	90	95
6–23 mo	1.0	—	—
12–23 mo	—	75	85
No breastfeeding			
0–5 mo	10.53	—	—
6–23 mo	2.28	—	—

a All interventions are applied to the 1–59 mo age group except ORS, which is applied to 0–59 mo and vitamin A, which is applied to 6–59 mo.

b Applied to nondysentery diarrheal deaths assumed to be 95% of total diarrheal deaths.

c Applied to dysentery diarrheal deaths assumed to be 5% of total diarrheal deaths.

d Applied to rotavirus deaths assumed to be 39% of total diarrheal deaths.

RR, relative risk.

### Estimating the Cost of Scale-up

We conducted a cost analysis for the specified interventions in both scale-up scenarios using an ingredients-based approach. For preventive interventions the population in need was defined as the number of children under 5 (vitamin A, rotavirus vaccine, breastfeeding) or, for the water, sanitation, and hygiene (WASH) interventions, the households with children under 5 y. For the treatment interventions we assumed that all cases would receive ORS and zinc; we also estimated the proportion of episodes that would meet the clinical definition of dysentery and require antibiotic treatment. Drug, supply and personnel time requirements per average case were calculated based on WHO treatment guidelines and expert opinion, and then costed using UNICEF's supply catalogue [21] and WHO CHOICE's country-specific database of medical staff salaries [22]. We also included costs of outreach activities and communication strategies. For non-WASH interventions, the analysis looked only at direct costs of providing the interventions; costs associated with capital investments such as building new health centers and training facilities are not captured in this analysis. We calculated the costs for the continuation of baseline coverage rates and the scale-up of both scenarios for all 68 countries included in this model. We then calculated the total per capita (total population) cost as well as the additional per capita cost to achieve each final coverage target for each year through 2015. Costs for the WASH interventions were kept separate from the individual-level prevention and treatment interventions.

## Results

**Figure 1:**
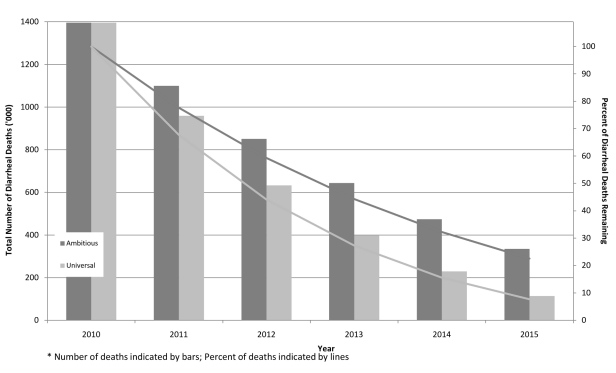
Trends in number and proportion of diarrheal deaths, under ambitious and universal scale-up plans.

Figure 1 presents trends in the number of diarrheal deaths between 2010 and 2015 for each of the two scenarios; it also shows the proportion of diarrheal deaths that would occur each year relative to the 2010 baseline values. Under the ambitious scenario, the number of diarrheal deaths would decline from more than 1.39 million a year in the baseline year of 2010 to 334,000 in 2015, which represents a 78% decline and nearly 1 million deaths averted in 2015. Over the 5-y scale-up period, more than 3.8 million deaths would be averted. Assuming linear scale-up, the estimated additional cost to achieve this reduction is US$0.49 per capita in 2015 for the non-WASH interventions and an additional US$1.78 per capita if 100% of the cost of the WASH interventions was to be borne by the public health system (Table 3). The total additional cost for scaling up all non-WASH interventions as per the ambitious scenario in these 68 countries over a 5-y period is US$7.7 billion. If WASH interventions were to be included the costs would rise to US$49.2 billion.

**Table 3:** Additional cost per capita to achieve targeted coverage rates for the ambitious and universal scale-up scenarios by 2015.

Year	Baseline Cost Per Capita (US$)^a^	Additional Cost Per Capita (US$)			
		Ambitious Scale-Up		Universal Scale-Up	
		All Interventions Excluding WASH^b^	WASH Interventions Alone^c^	All Interventions Excluding WASH^b^	WASH Interventions Alone^c^
2011	2.77	0.11	1.38	0.17	2.43
2012	2.71	0.21	1.58	0.33	2.63
2013	2.68	0.30	1.68	0.49	2.86
2014	2.63	0.40	1.75	0.65	3.04
2015	2.57	0.49	1.78	0.80	3.24
2011–2015	NA	1.52	8.18	2.47	14.23

a Assumes maintaining 2010 coverage levels through 2015.

b Interventions include vitamin A, rotavirus vaccine, and breastfeeding for prevention and ORS, zinc, and antibiotics for dysentery for treatment of diarrhea.

c Interventions include hand-washing, improved sanitation, access to safe water, and home purification of water.

NA, not available.

**Figure 2:**
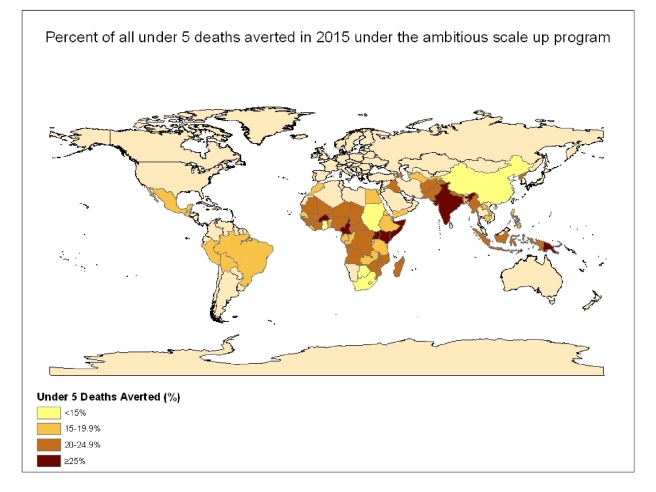
Number of child deaths averted in 2015 under the universal scale-up program for the 68 countries included in the analysis.

With the universal coverage scenario, the number of diarrheal deaths would drop to less than 115,000 in 2015, more than a 92% decline from the 2010 levels, representing nearly 1.4 million deaths averted in 2015 (Figures 1 and 2) and more than 4.9 million deaths during the 5-y scale-up period. To achieve these coverage rates, we estimate an additional cost of US$0.80 per capita in 2015 and an additional US$3.24 per capita with the addition of all WASH interventions at the highest coverage rates and all costs borne by the health system (Table 2). The total additional cost for scaling up all non-WASH interventions, as per the universal scenario in the 68 countries included in these analyses over a 5-y period, is US$12.5 billion. If WASH interventions are also included the costs rise to US$84.8 billion. Under both scenarios, 51% of deaths averted would be in just five countries: India, Nigeria, Democratic Republic of the Congo, Pakistan, and Afghanistan, that is 700,000 and 900,000 deaths in 2015 in the ambitious and universal scale-up scenarios, respectively.

## Discussion

In this exercise we used *LiST* to simulate the potential lives that could be saved by scaling-up ten simple and effective interventions for the prevention and treatment of diarrhea in 68 priority countries. The results of this modeling exercise demonstrate that with currently available technology, diarrheal deaths could be reduced by at least 78% by the end of 2015. To reach MDG4 by 2015, the number of child deaths needs to be reduced by an additional 4.6 million annually from the 2008 estimate of 8.8 million. Reducing diarrheal deaths by more than 1.4 million per year would be a major contribution toward this goal.

By using *LiST* to model the impact of scaling up multiple interventions we are able to illustrate the potential benefits of two scale-up scenarios over a 5-y period. *LiST* is a useful tool for modeling the effect of multiple prevention and treatment interventions because the effect of each intervention is applied step-wise in a cohort model. With this type of model, the effect of a second intervention is only observed on the residual of the first intervention. This ensures that potential lives are not saved more than once thus eliminating the risk of double counting. While coverage for many interventions, such as ORS, is routinely collected in most countries, coverage data for water and sanitation interventions that truly reflect optimal practices are extremely limited and in the case of home purification of water, completely missing for many countries. Thus, as coverage data improve, estimates generated by *LiST* will continue to improve.

In this exercise we did not account for the fact that a proportion of children will have access to all interventions, while a proportion of children representing those hardest to reach will likely receive few, if any, of the interventions [23]. The *LiST* tool assumes linearity between coverage of the intervention and the lives saved. Though we recognize that this may not be an accurate portrayal of what happens as coverage increases within a community, we do not currently have the evidence to support an alternative such as a threshold effect, minimum coverage, or herd immunity for these interventions. Ensuring that these lifesaving diarrhea prevention and treatment interventions reach the poorest and most vulnerable populations will be crucial for achievement of equity and maximum impact when scaling up programs if the predicted mortality reductions are to be achieved. For this model, the baseline scenario assumes current coverage values would remain the same through 2015 and thus diarrhea mortality rates would also remain constant. We recognize that there might be some small change in diarrhea mortality as a result of improved economic conditions, and other factors within a community which could also impact diarrhea mortality and are not captured here, but we believe these changes would be relatively small over the short time period captured in this exercise. This limitation could thus be more problematic over a longer time period where the magnitude of these societal changes could be expected to be much greater.

In this analysis, we estimated the cost of scaling up selected interventions in 68 countries. At an additional cost of US$0.80 per person per year, we can achieve nearly universal coverage of many key diarrhea prevention and treatment interventions. With additional investments in water and sanitation by households and the public sector we could ensure nearly every young child has access to safe water. We did not calculate costs per life saved because this analysis presents only the diarrheal deaths averted and thus fails to capture the full impact. Many of the diarrhea interventions avert deaths from other causes, either directly though prevention of pneumonia (e.g. breastfeeding) or indirectly via a reduction in diarrhea incidence (e.g. hygiene promotion) and stunting and thus the diarrheal deaths averted, which are presented here, likely underestimate the total deaths averted when scaling up this package of interventions.

It was beyond the scope of this analysis and costing exercise to fully estimate the costs required to adequately strengthen the health system in 68 diverse countries to sustainably maintain these high coverage levels. Furthermore, evidence suggests that cost curves are complex and nonlinear for infrastructure, commodities management, transportation, performance monitoring, and supervision [24]. We recognize that ensuring coverage in geographically or socially underserved communities may require strategies or delivery channels that are more costly than those needed to reach more accessible populations and, thus, generalizing per capita expenses where both disease burden and access to the health system vary has its limitations [25],[26]. Additional studies to explore variations in costing strategies are needed and may produce results that are more tailored to specific countries and populations.

To ensure the high coverage rates proposed here are achievable, new resources are needed to strengthen health systems for delivery of services, and to support the introduction and scale-up of recently introduced interventions for diarrhea treatment (low osmolarity ORS and zinc) and prevention (rotavirus vaccine) [27]. It is recognized that intense promotion of ORS use at the community level, and training of health workers during the WHO program for the control of diarrheal disease in the 1980s, was successful in scaling up coverage and reducing diarrheal deaths, although progress stagnated during the 1990s [3],[28]. There is evidence that promotion of zinc for diarrhea treatment alongside ORS can increase uptake and use, reduce unnecessary antibiotic use, and reinvigorate community management of diarrhea [29],[30]. However, there are a number of potential obstacles related to financing, national policy formulation, training, service delivery, and demand creation that are currently limiting scale-up of these strategies and that require urgent attention.

Real progress can be made if the prevention and treatment of diarrhea becomes an international priority and the global health community commits to a number of key actions as laid out in the 2009 UNICEF and WHO report [3]: mobilizing dedicated and sufficient funding for diarrhea control; leveraging global partnerships and networks for strong and effective advocacy; establishing clear and targeted health promotion and behavior change communication strategies; expanding the reach of health services into communities to ensure that diarrhea prevention and treatment is a central component of a “revitalized” community-based primary health care approach; and undertaking complementary efforts across both public and private sectors to promote innovations in supply and delivery of these key interventions to reach high and equitable coverage. In addition, because increasing coverage of these interventions requires input and leadership from multiple sectors and ministries within government, coordination will be critical to ensure success. lf the bottlenecks can be overcome and the international community can collectively deliver on the key actions noted above, then child morbidity and mortality due to diarrhea can be dramatically reduced and contribute to the achievement of MDG4. These analyses remind us that reaching goals in reducing under-five mortality does not require the development of new technologies or interventions; rather, these can be reached by implementing existing low cost and effective interventions.

## Supporting Information

Table S1 Web appendix.
(0.04 MB XLSX)

## Abbreviations

JMP: Joint Monitoring Program
*LiST*: Lives Saved Tool
ORS: oral rehydration salts
WASH: water, sanitation, and hygiene
